# Publisher Correction: Ultracompact mirror device for forming 20-nm achromatic soft-X-ray focus toward multimodal and multicolor nanoanalyses

**DOI:** 10.1038/s41467-024-46716-8

**Published:** 2024-03-14

**Authors:** Takenori Shimamura, Yoko Takeo, Fumika Moriya, Takashi Kimura, Mari Shimura, Yasunori Senba, Hikaru Kishimoto, Haruhiko Ohashi, Kenta Shimba, Yasuhiko Jimbo, Hidekazu Mimura

**Affiliations:** 1 School of Engineering, The University of Tokyo, 7-3-1 Hongo, Bunkyo, Tokyo 113-8656 Japan; 2 Japan Synchrotron Radiation Research Institute, 1-1-1 Koto, Sayo, Sayo District, Hyogo, 679-5198 Japan; 3 The Institute for Solid State Physics, The University of Tokyo, 5-1-5 Kashiwanoha, Kashiwa, Chiba 277-8581 Japan; 4 RIKEN SPring-8 Center, 1-1-1 Koto, Sayo, Sayo District, Hyogo 679-5148 Japan; 5 Department of Refractory Viral Infection, Research Institute, National Center for Global Health and Medicine, 1-21-1 Toyama, Shinjuku, Tokyo 162-8655 Japan; 6 Research Center for Advanced Science and Technology, The University of Tokyo, 4-6-1 Komaba, Meguro, Tokyo 153-8904 Japan

**Keywords:** X-rays, Molecular neuroscience, Scanning probe microscopy, Nanometrology

Correction to: *Nature Communications* 10.1038/s41467-023-44269-w, published online 07 February 2024

The original version of this Article contained an error in Fig. 1, in which the resolution of Figure 1b and 1c is low. This has been corrected in both the PDF and HTML versions of the Article.

