# Supplementary material for: Ultracompact mirror device for forming 20-nm achromatic soft-X-ray focus toward multimodal and multicolor nanoanalyses
Source: Nat Commun. 2024 Feb 7;15:665. doi: 10.1038/s41467-023-44269-w (PMC10850520; doi:10.1038/s41467-023-44269-w)
Supplement: Supplementary file 1 — Supplementary Information [file 41467_2023_44269_MOESM1_ESM.pdf]

Supplementary information for "Ultracompact mirror device for forming 20-nm achromatic soft-X-ray focus toward multimodal and multicolor nanoanalyses"

Takenori Shimamura[1,2,3*], Yoko Takeo[2,3], Fumika Moriya[1], Takashi Kimura[3], Mari Shimura[4], Yasunori Senba[2,5], Hikaru Kishimoto[2], Haruhiko Ohashi[2,5], Kenta Shimba[1], Yasuhiko Jimbo[1] and Hidekazu Mimura[5,6*]

[1*]School of Engineering, The University of Tokyo, 7-3-1 Hongo, Bunkyo, Tokyo 113-8656 Japan.
[2]Japan Synchrotron Radiation Research Institute, 1-1-1 Koto, Sayo, Sayo District, Hyogo 679-5198 Japan.
[3]The Institute for Solid State Physics, The University of Tokyo, 5-1-5 Kashiwanoha, Kashiwa, Chiba 277-8581 Japan.
[4]Department of Refractory Viral Infection, Research Institute, National Center for Global Health and Medicine, 1-21-1 Toyama, Shinjuku, Tokyo 162-8655 Japan.
[5]RIKEN SPring-8 Center, 1-1-1 Koto, Sayo, Sayo District, Hyogo 679-5148 Japan.
[6]Research Center for Advanced Science and Technology, The University of Tokyo, 4-6-1 Komaba, Meguro, Tokyo 153-8904 Japan.

*Corresponding author(s). E-mail(s):
tshimamura@issp.u-tokyo.ac.jp;
mimura@upm.rcast.u-tokyo.ac.jp;

1 Mathematical derivation of diffracted focus wavefields

From a geometrical viewpoint, a shorter focal length alleviates the effects of surface imperfections on ray deflection, as shown in Fig. 7. Figure errors cause an uneven slope distribution, which scatters rays in proportion to the focal length.

Wave optics shows that the diffraction triggered by practical figure errors increases with focal length. Light focused at $z = f$ with a focusing device at $z = 0$ can be modeled as a wavefield traveling along the positive z-direction from an aperture lying in the (x, y) plane to the focal area across the (u, v) plane, as shown in Fig. 1a. This wavefield propagation is expressed by the Fresnel diffraction formula [1]:

$$U_\mathrm{f}(u,v) = \frac{1}{j\lambda f} \exp\left\{jkf\left(\frac{u^2+v^2}{2f^2}\right)\right\} \iint_{-\infty}^{\infty} \left[U_\mathrm{a}(x,y) \exp\left\{j\frac{k}{2f}(x^2+y^2)\right\}\right] \exp\left\{-j\frac{2\pi}{\lambda f}(ux+vy)\right\} \mathrm{d}x\mathrm{d}y \quad , \tag{1}$$

where $j = \sqrt{-1}$, λ is the wavelength of the monochromatic light, $k = 2\pi/\lambda$ is the wavenumber, f is the focal length, and U_f and U_a are the focus and aperture wavefields, respectively. Focusing devices transform the incident wavefront into a spherical shape that converges at the focus. The spherical wavefront cancels the quadratic phase factor within the integral in Supplementary Eq. (1), automatically satisfying the Fraunhofer diffraction formula [1]:

$$U_\mathrm{f}(u,v) = \frac{1}{j\lambda f} \exp\left\{jkf\left(\frac{u^2+v^2}{2f^2}\right)\right\} \iint_{-\infty}^{\infty} U_\mathrm{a}(x,y) \exp\left\{-j\frac{2\pi}{\lambda f}(ux+vy)\right\} \mathrm{d}x\mathrm{d}y \quad . \tag{2}$$

The Fourier transform of $U_\mathrm{a}(x, y)$ defined by

$$F_\mathrm{a}(f_X, f_Y) = \mathcal{F}[U_\mathrm{a}] = \iint_{-\infty}^{\infty} U_\mathrm{a}(x,y) \exp\{-j2\pi(f_X x + f_Y y)\} \mathrm{d}x\mathrm{d}y \tag{3}$$

simplifies Supplementary Eq. (2) to [1]

$$U_\mathrm{f}(u,v) = A(u,v;f) F_\mathrm{a}\left(\frac{u}{\lambda f}, \frac{v}{\lambda f}\right) \quad , \tag{4}$$

where

$$A(u,v;f)=\frac{1}{j\lambda f}\exp\left\{jkf\left(\frac{u^2+v^2}{2f^2}\right)\right\}=A_X(u;f)A_Y(v;f) \quad . \tag{5}$$

When the exponential coefficient $A(u,v;f)$ is neglected, Supplementary Eq. (4) indicates that the wavefield at the focus spatially magnifies the Fourier transform of the aperture wavefield by a factor of λ and f [1].

The ideal spherical wavefront at the aperture yields a focus size that is independent of f if the numerical aperture (NA) is constant and thus the aperture size is scaled by f as well. For instance, because a combination of plane X-ray mirrors limits the incident wavefield $U_{\rm i}(x,y,z)$ to a square shape, this aperture can be expressed by

$$U_{\rm a}^{\rm ideal}(x,y)=U_{\rm i}(x,y)\times \mathrm{rect}\left(\frac{x}{a}\right)\times \mathrm{rect}\left(\frac{y}{b}\right) \quad , \tag{6}$$

where $2a$ and $2b$ are the aperture dimensions in the x- and y-directions, respectively, and $U_{\rm i}$ is the incident wavefield, which is normally uniform. $\mathrm{rect}(x)$ is a rectangle function defined as

$$\mathrm{rect}(x)=\begin{cases}1 & (|x|< 0.5)\\ 0.5 & (|x|= 0.5)\\ 0 & (\text{otherwise}) \quad .\end{cases} \tag{7}$$

As each mirror focuses X-rays in a single direction, it is assumed that the wavefield can be written as a product of two separate functions:

$$U_{\rm f}(u,v)=U_{{\rm f}X}(u)U_{{\rm f}Y}(y) \quad . \tag{8}$$

This assumption, which reduces the present problem to one direction, agrees with the form of Supplementary Eq. (3-6) and the experimental results for X-ray diffractive focusing devices [2, 3]. Substituting Supplementary Eq. (6) into Supplementary Eq. (4) yields the following wavefield at the mirror focus location:

$$\begin{aligned}U_{{\rm f}X}(u)&=A_X(u;r)\left[\mathcal{F}[U_{\rm i}]*\mathcal{F}\left[\mathrm{rect}\left(\frac{x}{a}\right)\right]\right]\left(\frac{u}{\lambda r}\right)\\ &=A_X(u;r)\left[\mathcal{F}[U_{\rm i}]\left(\frac{u}{\lambda r}\right)*\left\{a\frac{\sin\left(\frac{\pi u a}{\lambda r}\right)}{\frac{\pi u a}{\lambda r}}\right\}\right] \quad ,\end{aligned} \tag{9}$$

where the symbol $*$ denotes the convolution operation. Here, the aperture plane is defined at the downstream edge of the grazing-incidence mirror and f is replaced by r. This aperture configuration disregards minor diffraction immediately after the reflection. Provided that the NA is constant at α, namely

$\arctan(a/r) \approx a/r \approx \alpha$ in the grazing-incidence configuration, Supplementary Eq. (9) becomes

$$U_{fX}(u) = A_X(u;r)r\alpha \left[\mathcal{F}[U_i] \left(\frac{u}{\lambda r} \right) * \left\{ \frac{\sin\left(\pi \frac{\alpha u}{\lambda}\right)}{\pi \frac{\alpha u}{\lambda}} \right\} \right] \quad . \tag{10}$$

The ideal mirror preserves the incident uniform wavefront at the aperture. The Fourier transform of such wavefields is the Dirac delta function, which is independent of λ and r, as in

$$\mathcal{F}[U_i] \left(\frac{u}{\lambda r} \right) = \delta \left(\frac{u}{\lambda r} \right) = \begin{cases} 1 & (u = 0) \\ 0 & (\text{otherwise}) \end{cases} \quad . \tag{11}$$

The convolution operation in Supplementary Eq. (10) results in the latter sinusoidal part being placed at the origin of the focal plane. Therefore,

$$U_{fX}(u) = A_X(u;r)r\alpha \left\{ \frac{\sin\left(\pi \frac{\alpha u}{\lambda}\right)}{\pi \frac{\alpha u}{\lambda}} \right\} \quad . \tag{12}$$

The coefficient depends on r as a larger aperture can accept more X-rays. The remaining part is independent of r and the intensity $|U_{fX}|^2$ produces a focus size that is independent of focal length.

However, X-ray mirrors have surface defects that are not negligible on the scale of X-ray wavelengths. The disturbance of the incident wavefront by these defects can be modeled as

$$\begin{aligned} U_a^{\text{error}}(x,y) &= P_m(x,y)U_a^{\text{ideal}}(x,y) \\ &= P_m(x,y)U_i(x,y)\text{rect}\left(\frac{x}{a}\right)\text{rect}\left(\frac{y}{b}\right) \quad . \end{aligned} \tag{13}$$

The mirror production process often leaves periodic figure errors in a single direction, as illustrated in Fig. 7. This allows the phase modulation factor $P_m(x,y)$ to be expressed by $P_{mX}(x,y) \times P_{mY}(x,y)$. A wavefront error at the aperture is determined by the height of the figure error h and the grazing angle θ [4]:

$$P_{mX}(x) = \exp\left[j\frac{2\pi}{\lambda} \left\{ 2h(\xi)\sin(\theta(\xi)) \right\} \right] \quad , \tag{14}$$

where $\xi(x)$ is the location on the mirror at which the incident X-rays are reflected before reaching the aperture location x. Whereas the range of x and ξ scales with r, the periodicity of $P_{mX}(x)$ does not depend on r because the spatial wavelength of the figure error is independent of mirror length. Supplementary Eq. (10) is modified as

$$U_{fX}(u) = A_X(u;r)r\alpha \left[\mathcal{F}[P_{mX} \times U_i] \left(\frac{u}{\lambda r} \right) * \text{sinc}\left(\frac{\alpha u}{\lambda} \right) \right] \quad , \tag{15}$$

where

$$\mathrm{sinc}(x) = \frac{\sin \pi x}{\pi x} \quad . \tag{16}$$

The convolution operation in Supplementary Eq. (15) displaces the origin of the sinc function or splits the original sinc function into several sinc-based functions. The value of $P_{\mathrm{m}X} U_{\mathrm{i}}$ does not depend on r or a, which cancels the introduced $1/(\lambda r)$ factor; therefore, the distribution of the sinc function centers is magnified by $1/(\lambda r)$. Supplementary Eq. (15) suggests that the diffraction due to figure errors generally scales with focal length.

To specify the distribution of the sinc function centers, Supplementary Eq. (14) can be simplified if the grazing angle has a sufficiently small value and a sufficiently small variation on the mirror surface. Such grazing-incidence mirrors can be presumed to satisfy

$$\begin{cases} \theta(\xi) \approx \theta_0 & \\ \xi(u) \approx \dfrac{x}{\sin \theta_0} & (-a < x < a) \\ h(\xi) = h_0 \cos \dfrac{2\pi}{d_{\mathrm{m}}} \xi \approx h_0 \cos \dfrac{2\pi}{d_{\mathrm{m}}} \dfrac{x}{\sin \theta_0} \quad , & \end{cases} \tag{17}$$

where θ_0 and h_0 are constant. d_{m} is the spatial wavelength for a mirror figure error. $d_{\mathrm{m}} \sin \theta_0$ is taken to be the wavelength projected to the aperture. Under the assumption that h_0 is sufficiently small, applying the Taylor expansion to Supplementary Eq. (14) yields

$$\begin{aligned} P_{\mathrm{m}X}(x) &\approx \exp\left[j \frac{2\pi}{\lambda} \left\{ 2(\sin \theta_0) h_0 \cos\left(\frac{2\pi}{d_{\mathrm{m}} \sin \theta_0} x \right) \right\} \right] \\ &\approx 1 + j \frac{2\pi}{\lambda} h_{\mathrm{a}} \cos\left(\frac{2\pi}{d_{\mathrm{a}}} x \right) - \frac{2\pi^2}{\lambda^2} h_{\mathrm{a}}^2 \cos^2\left(\frac{2\pi}{d_{\mathrm{a}}} x \right) \\ &= \left(1 - \frac{\pi^2 h_{\mathrm{a}}^2}{\lambda^2} \right) - \frac{\pi^2 h_{\mathrm{a}}^2}{\lambda^2} \cos\left(\frac{4\pi}{d_{\mathrm{a}}} x \right) + j \frac{2\pi h_{\mathrm{a}}}{\lambda} \cos\left(\frac{2\pi}{d_{\mathrm{a}}} x \right) \quad , \end{aligned} \tag{18}$$

where $h_{\mathrm{a}} = 2h_0 \sin \theta_0$ and $d_{\mathrm{a}} = d_{\mathrm{m}} \sin \theta_0$. Its Fourier transform is

$$\begin{aligned} \mathcal{F}[P_{\mathrm{m}X} \times U_{\mathrm{i}}]\left(\frac{u}{\lambda r} \right) = &\left(1 - \frac{\pi^2 h_{\mathrm{a}}^2}{\lambda^2} \right) \delta(u) \\ &- \frac{\pi^2 h_{\mathrm{a}}^2}{2\lambda^2} \left\{ \delta\left(u - \frac{2\lambda r}{d_{\mathrm{a}}} \right) + \delta\left(u + \frac{2\lambda r}{d_{\mathrm{a}}} \right) \right\} \\ &+ j \frac{\pi h_{\mathrm{a}}}{\lambda} \left\{ \delta\left(u - \frac{\lambda r}{d_{\mathrm{a}}} \right) + \delta\left(u + \frac{\lambda r}{d_{\mathrm{a}}} \right) \right\} \quad . \end{aligned} \tag{19}$$

Supplementary Eq. (19) is divided into three terms. The first term depends on only u. The latter two terms are non-zero at $u = \pm \lambda r / d_{\mathrm{a}}$ or $\pm 2\lambda r / d_{\mathrm{a}}$

(these values depend on r). If Supplementary Eq. (19) is substituted into Supplementary Eq. (15), the convolution operation splits the sinc function into $u = 0, \pm\lambda r/d_\mathrm{a}, and \pm 2\lambda r/d_\mathrm{a}$, namely

$$\begin{aligned} U_{\mathrm{f}X}(u) \approx A_X(u;r)r\alpha\Bigg\{&(1-\phi^2)\mathrm{sinc}\left[\frac{\alpha u}{\lambda}\right] \\ &-\frac{\phi^2}{2}\left(\mathrm{sinc}\left[\frac{\alpha}{\lambda}\left(u-\frac{2\lambda r}{d_\mathrm{a}}\right)\right]+\mathrm{sinc}\left[\frac{\alpha}{\lambda}\left(u+\frac{2\lambda r}{d_\mathrm{a}}\right)\right]\right) \\ &+j\phi\left(\mathrm{sinc}\left[\frac{\alpha}{\lambda}\left(u-\frac{\lambda r}{d_\mathrm{a}}\right)\right]+\mathrm{sinc}\left[\frac{\alpha}{\lambda}\left(u+\frac{\lambda r}{d_\mathrm{a}}\right)\right]\right)\Bigg\} \quad , \end{aligned} \tag{20}$$

where $\phi = \pi h_\mathrm{a}/\lambda$. These sinc functions express auxiliary focus wavefields, which contribute to the intensity. If the surface has no periodic figure errors, namely $d_\mathrm{a} \to \infty$, Supplementary Eq. (20) reduces to

$$U_{\mathrm{f}X}(u) \approx A_X(u;r)r\alpha(1-2\phi^2+j2\phi)\mathrm{sinc}\left[\frac{\alpha u}{\lambda}\right] \quad . \tag{21}$$

Here, as $\exp(j2\phi) \approx 1 + j2\phi - 2\phi^2$, Supplementary Eq. (21) shows the effect of the uniform phase shift 2ϕ at the aperture. When $h_\mathrm{a} = 2h_0 \sin\theta_0 \to 0$, Supplementary Eq. (21) concurs with the ideal focus wavefield in Supplementary Eq. (12).

The offset between the auxiliary focus wavefields is

$$\Delta u = \frac{\lambda r}{d_\mathrm{a}} = \frac{\lambda r}{d_\mathrm{m}\sin\theta_0} \approx \frac{\lambda r}{d_\mathrm{m}\theta_0} \quad . \tag{22}$$

Supplementary Eq. (22) can be interpreted as the interference fringe pitch on the focal plane that is generated by a virtual transmission grating at the aperture. The interference fringe pitch can be deduced using the periodic figure error projected onto the aperture and the optical path difference between the neighboring error peaks. Whereas the intensity of the diffracted focus sidelobes could be suppressed compared to the main lobe, Supplementary Eq. (22) indicates that the periodic figure errors cause extra focus points, which spread with the focal length, as suggested by Supplementary Eq. (15). A short-focal-length mirror (see Fig. 7b) can suppress the effect of the figure errors and concentrate the reflected X-rays.

If $\lambda = 1.24$ nm (1-keV photon energy), $r = 1$ mm, $d_\mathrm{m} = 500$ µm, and $\theta_0 = 25$ mrad, Δu becomes 105 nm. A longer focal length spreads this diffracted focus area more widely. Additionally, Supplementary Eq. (22) suggests the following spatial wavelength range for the figure error that may compromise the focus size:

$$\begin{aligned} \frac{\Delta u_\mathrm{max}}{2} &> \Delta u \approx \frac{\lambda r}{d_\mathrm{m}\theta_0} \\ d_\mathrm{m} &> \frac{2\lambda r}{\Delta u_\mathrm{max}\theta_0} \quad . \end{aligned} \tag{23}$$

This means that the mirror surface for the focus size Δu_{max} is required to be approximately flat in the spatial wavelength range shorter than $2\lambda r/(\Delta u_{\mathrm{max}}\theta_0)$.

Supplementary Eq. (15) can also explain X-rays diffracted by reflective gratings. For simplicity, the wavefront error $P_{\mathrm{m}X}(x)$ replicated from the device surface via Supplementary Eq. (14) is assumed to be $\exp(j2\pi x/d_{\mathrm{a}})$ with a spatial wavelength of d_{a} at the aperture. This wavefront design can be used in blazed varied-space plane gratings [5], total-reflection zone plates [6, 7], or Bragg-Fresnel lenses [3, 8]. The smooth incident wavefront results in

$$\mathcal{F}[P_{\mathrm{m}X} \times U_{\mathrm{i}}]\left(\frac{u}{\lambda r}\right) = \delta\left(\frac{u}{\lambda r} - \frac{1}{d_{\mathrm{a}}}\right) \quad . \tag{24}$$

When $d_{\mathrm{a}} = \infty$, Supplementary Eq. (24) is identical to Supplementary Eq. (11). The convolution operation in Supplementary Eq. (15) displaces the origin of the sinusoidal part to $u = \lambda r/d_{\mathrm{a}}$, resulting in

$$U_{\mathrm{f}X}(u) = A_X(u;r)r\alpha\left\{\frac{\sin\left(\frac{\pi\alpha}{\lambda}(u - \frac{\lambda r}{d_{\mathrm{a}}})\right)}{\frac{\pi\alpha}{\lambda}(u - \frac{\lambda r}{d_{\mathrm{a}}})}\right\} \quad . \tag{25}$$

According to Supplementary Eq. (25), the diffraction caused by the phase modulation makes the focus wavefield centered at $u = \lambda r/d_{\mathrm{a}}$ and the aperture generates interference fringes spaced at $u = \alpha/\lambda$. The former value scales with r and agrees with the design principle of reflective concave gratings or total-reflection zone plates. Whereas Supplementary Eq. (25) shows a concentrated wavefield at $u = \lambda r f_0$, the grating shape superposed by multiple spatial wavelengths d_{a} scatters the center of the auxiliary focus wavefields, as found in the mirror figure errors.

2 Sample specifications

2.1 Inorganic specimens

The inorganic specimens included a 10-µm-diameter pinhole for knife-edge scanning, a pattern for ptychography with the letters "SP8" spanning an area of 2×3 µm^2, and scattered ϕ100-nm Au nanoparticles. All items were produced on a transmission electron microscopy (TEM) grid (PELCO Silicon Nitride Support Films for TEM, TED PELLA, Inc.), which was a 3-mm-diameter Si disk with nine 200-nm-thick silicon nitride supporting film windows, as shown in Supplementary Fig. 1. To reduce X-ray transmission outside the pinhole and pattern, the silicon nitride supporting film was first coated with a 300-nm-thick Ni layer via metal evaporation, and then a focused ion beam (FIB) system (FIB-SEM SII XVision 200 TB, Hitachi High-Tech Corporation) was utilized to fabricate the objects on the Ni-coated windows. A commercially available ϕ100-nm Au nanoparticle buffer (Gold Colloid, BBI Solutions) was

added to a solution mixture of ultra-purified water and ethanol in a 1:1:1 ratio. The solution was dropped onto the pristine silicon nitride supporting film and then air-dried. The amount of the dropped solution was varied to find the optimal distribution of nanoparticles on the film. The position and diameter of Au nanoparticles were determined using scanning electron microscopy (SEM) (FE-SEM Hitachi Regulus8230, Hitachi High-Tech Corporation) beforehand.

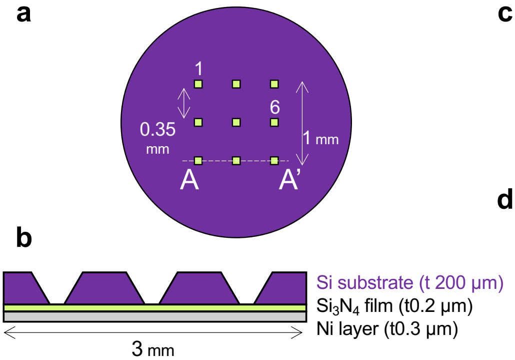

Supplementary Fig. 1 Knife-edge and letter objects fabricated on supporting film windows. **a** Schematic top view of 3-mm-diameter Si disk with nine 200-nm-thick silicon nitride supporting film windows. **b** Schematic cross-section view at A-A' line. **c** Position of 10-μm-diameter pinhole for knife-edge scanning within Si window frame. **d** Scanning electron microscopy (SEM) micrograph of 10-μm-diameter pinhole. The arrow indicates the circular area damaged during the focused ion beam fabrication process. Scale bar: 5 μm. **e** Location of letter pattern "SP8". **f** SEM micrograph of "SP8" letter pattern. Scale bar: 1 μm.

2.2 Hep G2 cell culture

RBRC-RCB01886 Hep G2 cells were obtained from the Riken Cell Bank of Japan (Tsukuba, Japan). Cells were maintained and plated using a previously described method [9]. Briefly, cells (3.2×10^3 per Si_3N_4 chip) were plated 21 hours before fixation. The cells were washed twice with PBS (-) containing 10% FBS and then once with PBS(-). The cells were fixed with 2% paraformaldehyde (#18814, ultra-pure EM grade; Polysciences Inc., Warrington, PA, USA) in PBS(-) for 10 minutes. The plates were washed with PBS(-) once and rinsed quickly with Milli-Q water three times, air-dried, and stored in clean tubes.

2.3 Culture of primary hippocampal neurons

The preparation of hippocampal cell cultures was performed using a previously described method [10]. Hippocampal cultures were prepared from Wistar rat brains at embryonic day 19. Hippocampal tissue was separated and dissected using HBSS. The isolated hippocampal cells were digested with a solution containing 0.5% trypsin (Life Technologies, CA, USA) for 15 minutes at 37 °C.

The addition of FBS-containing medium stopped digestion. Cells were plated on chip substrates treated with 0.1% polyethyleneimine (Wako, Japan) for 1 hour at 37 °C, followed by 20 µg/mL of laminin (Life Technologies, CA, USA) for 1 hour at 37 °C. The initial cell density was 500 cells/mm^2. Cells were cultured in primary culture medium containing Neurobasal Medium (Thermo Fisher Scientific, MA, USA), 2% B27 supplement (Thermo Fisher Scientific, MA, USA), 2 mM GlutaMAX (Thermo Fisher Scientific, MA, USA), and 1% penicillin/streptomycin (Thermo Fisher Scientific, MA, USA). Half of the culture medium was changed every three days. Cultures were maintained in a CO_2 incubator under 5% CO_2 at 37 °C in a water-saturated atmosphere. At 7 days after seeding, the hippocampal culture was fixed in 4% paraformaldehyde (FUJIFILM Wako Pure Chemical, Japan) and then dried at room temperature.

3 Results of achromatic soft-X-ray nanofocusing

3.1 Evaluation of focusing profiles

Supplementary Fig. 2 shows the focusing profiles evaluated using the knife-edge scanning method at photon energies of 0.3, 0.4, 0.6, and 1 keV. A Foucault knife-edge test showed that the focus spot remained within the Rayleigh range. The knife edge was therefore scanned at the same position. The knife-edge scanning was performed vertically and horizontally using the 10-µm-diameter pinhole fabricated on the Ni-coated silicon nitride supporting film. Supplementary Fig. 3 shows the ptychographically reconstructed focusing profiles. The focused beam intensity and the abrupt changes in the total photon count demonstrate that the incident X-rays were well focused into a small spot.

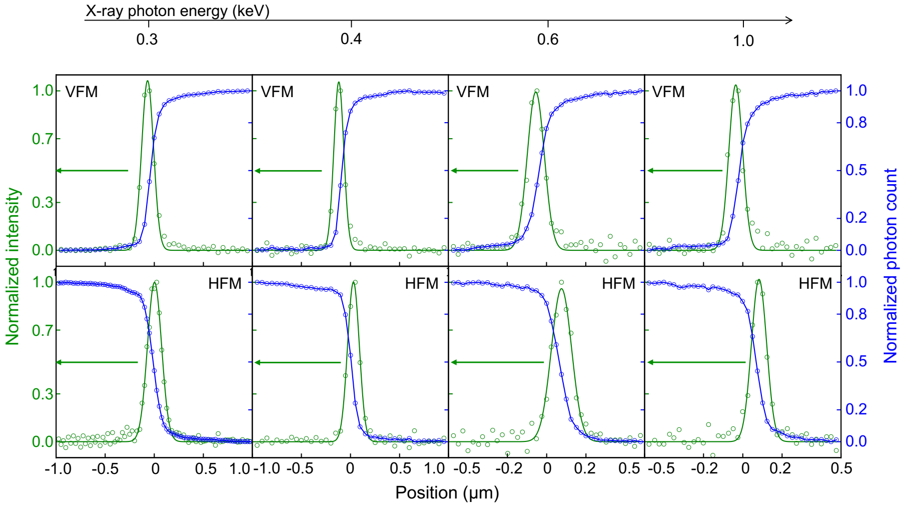

Supplementary Fig. 2 Focusing profiles evaluated using knife-edge scanning method.

Supplementary Fig. 3 shows the focusing profiles ptychographically reconstructed at photon energies of 0.3, 0.6, 1, and 2 keV. The letter pattern "SP8" was employed for 0.3, 0.6, and 1 keV, and ϕ100-nm Au nanoparticles were utilized for 2 keV. The employed grating-mirror pair was M21a-G3a for 0.3, 0.6, and 1 keV and M21a-G4a for 0.4 and 2 keV. Focus sizes were defined as the

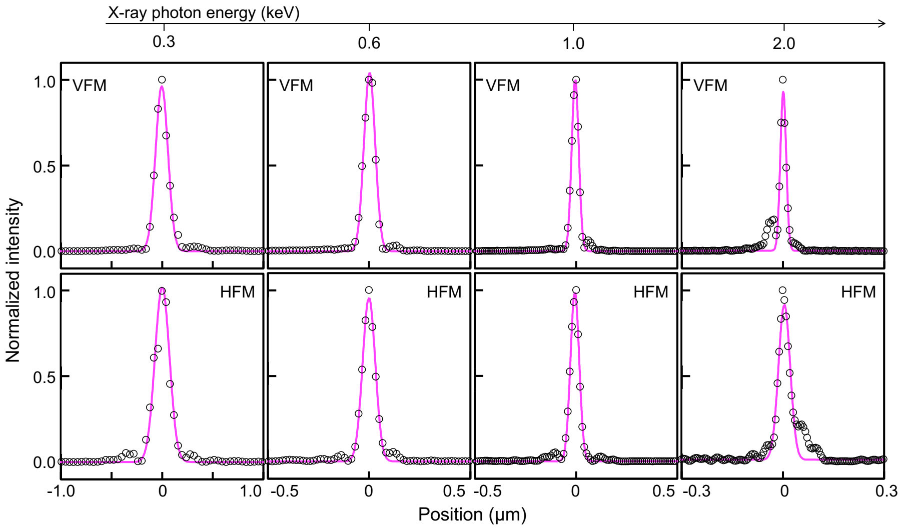

Supplementary Fig. 3 Focusing profiles evaluated using ptychography.

full width at half maximum (FWHM) values of Gaussian-fit profiles, which are listed in Supplementary Table 1. The ratio of the flux within the focus was defined as the number of photons within the range of the focus spot size over the total photon count.

3.2 Analyses of ptychographically reconstructed wavefronts

Supplementary Fig. 4 shows the figure errors for the vertically focusing mirror (VFM) and horizontally focusing mirror (HFM). The figure errors were evaluated using interferometry and ptychography. The ptychographically reconstructed probe functions were first propagated to the downstream end of the mirrors and the obtained wavefronts were then converted to figure errors. Two types of VFM figure errors are shown in Supplementary Fig. 4 because the mirror surface illuminated by X-rays was slightly shifted in the tangential direction after the mirror-grating pair was changed and the mirror posture was readjusted. Defocus causes spherical aberration of the wavefront. The ptychographically reconstructed vertical wavefront contained slight aberration at the sample position for photon energies of 0.3 and 2 keV. In this case, the wavefront was first regressed to obtain the best square polynomial. The square-removed

Supplementary Table 1 Focus size and flux ratio within focus spot. The percentage of the photon flux within the one-direction focus range is given in parentheses next to the focus size.

Method	Photon energy (keV)	Vertical focus size[a] (nm)	Horizontal focus size[a] (nm)	Percentage of flux within rectangular focus area (%)
Knife-edge scanning	0.3	142.5 (64)	169.0 (66)	42
	0.4	118.6 (62)	140.4 (65)	40
	0.6	103.3 (66)	121.5 (68)	45
	1	77.4 (61)	96.7 (65)	40
Ptychography	0.3	144.0 (68)	169.4 (65)	44
	0.6	65.3 (70)	73.4 (66)	46
	1	40.9 (67)	50.7 (61)	41
	2	20.4 (52)	40.7 (43)	22
Design value range	0.3	129.9 - 146.5	150.6 - 168.8	-
	0.4	97.4 - 109.9	112.2 - 126.6	-
	0.6	65.0 - 73.3	74.8 - 84.4	-
	1	39.0 - 44.0	44.9 - 50.6	-
	2	19.5 - 22.0	22.4 - 25.3	-

[a]Full width at half maximum values.

wavefront was then converted to the figure error [11]. For the other wavefronts, linear composition was removed and then the wavefronts were converted to the figure error. The ptychographically reconstructed figure errors are in good agreement with that evaluated by interferometry.

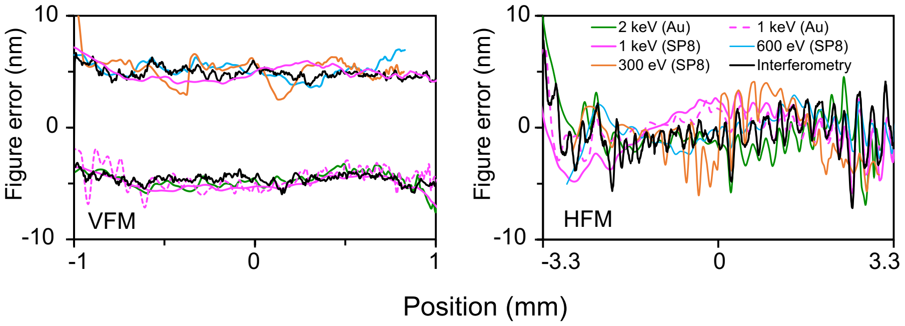

Supplementary Fig. 4 Figure errors for vertically focusing mirror (VFM) and horizontally focusing mirror (HFM) calculated from ptychographically reconstructed probe functions.

3.3 Focus position

A Foucault knife-edge test showed that the focus spot remained within the Rayleigh range. The knife edge was therefore scanned at the same position along with the X-ray path. The in-plane shifts were calculated from the center of the Gaussian-fit profiles. The small shifts of the focus position shown in Fig. 3a can stem from the transparency of the knife edge to high-energy X-rays. The secondary slits (S2 and SCa) and the monochromator were also moved to change photon energy; such movement of the upstream optics may have contributed to the drift in the focus spot and caused the mirror surface illuminated by X-rays to be shifted in the tangential direction.

The amount of defocus was evaluated by backpropagating ptychographically reconstructed wavefields to the downstream of the X-ray mirror and calculating the difference in the radius of curvature of the wavefields. Using the coordinate system shown in Fig. 7a, the circular wavefront that is ideally formed with a smooth mirror is expressed as a part of a circle:

$$x^2 + (z - r)^2 = r^2 \,. \tag{26}$$

The wavefront is located at the downstream side of the mirror, which usually has a small spatial acceptance compared to the working distance (i.e., $x/r \ll 1$).

$$\begin{aligned} z &= r \pm r\sqrt{1 - \frac{x^2}{r^2}} \\ &\approx 2r - \frac{x^2}{2r} \text{ or } \frac{x^2}{2r} \\ \therefore z &\approx \frac{x^2}{2r} \,. \end{aligned} \tag{27}$$

The defocus amount Δr is the same as the difference in the radius of curvature of the wavefront. If the coefficient of the squared term for the reconstructed wavefront is a,

$$\begin{aligned} z &= \frac{x^2}{2(r + \Delta r)} - \frac{x^2}{2r} \\ &= \frac{-\Delta r}{2(r + \Delta r)r} x^2 \\ &= a x^2 \,. \end{aligned} \tag{28}$$

Therefore,

$$\Delta r = \frac{-2r^2 a}{2ra + 1} \,. \tag{29}$$

References

[1] Joseph, G.W.: Introduction to Fourier Optics, Fourth edition edn. W.H. Freeman and Company, New York (2017)

[2] Leitenberger, W., Pietsch, U.: A monolithic Fresnel bimirror for hard X-rays and its application for coherence measurements. J. Synchrotron Radiat. **14**(2), 196–203 (2007). https://doi.org/10.1107/S0909049507003846

[3] Bonse, U., Riekel, C., Snigirev, A.A.: Kirkpatrick–Baez microprobe on the basis of two linear single crystal Bragg–Fresnel lenses. Rev. Sci. Instrum. **63**(1), 622–624 (1992). https://doi.org/10.1063/1.1142671

[4] Shi, X., Reininger, R., Sanchez Del Rio, M., Assoufid, L.: A hybrid method for X-ray optics simulation: Combining geometric ray-tracing and wavefront propagation. J. Synchrotron Radiat. **21**(4), 669–678 (2014). https://doi.org/10.1107/S160057751400650X

[5] Itou, M., Harada, T., Kita, T.: Soft x-ray monochromator with a varied-space plane grating for synchrotron radiation: design and evaluation. Appl. Opt. **28**(1), 146 (1989). https://doi.org/10.1364/ao.28.000146

[6] Takano, H., Tsuji, T., Hashimoto, T., Koyama, T., Tsusaka, Y., Kagoshima, Y.: Sub-15nm hard X-ray focusing with a new total-reflection zone plate. Appl. Phys. Express **3**(7), 3–6 (2010). https://doi.org/10.1143/APEX.3.076702

[7] Tsuji, T., Takano, H., Koyama, T., Tsusaka, Y., Kagoshima, Y.: Development of a total reflection zone plate for hard X-ray focusing. Jpn. J. Appl. Phys. **49** (2010). https://doi.org/10.1143/JJAP.49.030207

[8] Kuznetsov, S.M., Snigireva, I.I., Snigirev, A.A., Engström, P., Riekel, C.: Submicrometer fluorescence microprobe based on Bragg-Fresnel optics. Appl. Phys. Lett. **65**(7), 827–829 (1994). https://doi.org/10.1063/1.112244

[9] Tanaka, Y.K., Usuzawa, H., Yoshida, M., Kumagai, K., Kobayashi, K., Matsuyama, S., Inoue, T., Matsunaga, A., Shimura, M., Ruiz Encinar, J., Costa-Fernández, J.M., Fukumoto, Y., Suzuki, N., Ogra, Y.: Formation Mechanism and Toxicological Significance of Biogenic Mercury Selenide Nanoparticles in Human Hepatoma HepG2 Cells. Chem. Res. Toxicol. **34**(12), 2471–2484 (2021). https://doi.org/10.1021/acs.chemrestox.1c00231

[10] Moriya, F., Shimba, K., Kotani, K., Jimbo, Y.: Modulation of dynamics

in a pre-existing hippocampal network by neural stem cells on a microelectrode array. J. Neural Eng. **18**(4) (2021). https://doi.org/10.1088/1741-2552/ac1c88

[11] Shimamura, T., Takeo, Y., Kimura, T., Perrin, F., Vivo, A., Senba, Y., Kishimoto, H., Ohashi, H., Mimura, H.: Fabrication of ultrashort submeter-radius x-ray mirrors using dynamic stencil deposition with figure correction. Rev. Sci. Instrum. **94**(4) (2023). https://doi.org/10.1063/5.0135367
